# Analysis of the Vibration Characteristics of a Leaf Spring System Using Artificial Neural Networks

**DOI:** 10.3390/s22124507

**Published:** 2022-06-14

**Authors:** Mehmet Bahadır Çetinkaya, Muhammed İşci

**Affiliations:** 1 Department of Mechatronics Engineering, Faculty of Engineering, University of Erciyes, Kayseri 38039, Turkey; 2 Department of Mechatronics, University of Kayseri, Kayseri 38280, Turkey; muhammedisci@kayseri.edu.tr

**Keywords:** real-time vibration analysis, leaf spring systems, radial basis neural networks, cascade-forward back-propagation neural networks

## Abstract

The real-time vibrations occurring in a leaf spring system may cause undesirable effects, such as stresses, strains, deflections, and surface deformations over the system. In order to detect the most appropriate working conditions in which the leaf spring system will work more stably and also to design optimized leaf spring systems, these external effects have to be detected with high accuracy. In this work, artificial neural network-based estimators have been proposed to analyze the vibration effects on leaf spring systems. In the experimental studies carried out, the vibration effects of low, medium, and high-pressure values applied by a hydraulic piston on a steel leaf spring system have been analyzed by a 3-axial accelerometer. After the experimental studies, the Radial Basis Artificial Neural Network (RBANN) and Cascade-Forward Back-Propagation Artificial Neural Network (CFBANN) based nonlinear artificial neural network structures have been proposed to analyze the vibration data measured from the leaf spring system under relevant working conditions. The simulation results represent that the RBANN structure can estimate the real-time vibrations occurring on the leaf spring system with higher accuracy and reaches lower RMS error values when compared to the CFBANN structure. In general, it can be concluded that the RBANN and CFBANN network structures can successfully be used in the estimation of real-time vibration data.

## 1. Introduction

The leaf spring systems constituting an important part of the suspension system of vehicles are used to absorb the vibrations and reduce the effects of external forces. Higher structural damping properties, with respect to the other types of springs, are one of the most remarkable characteristics of these systems [1]. The leaf spring systems are exposed to external forces of different types and intensities, such as pressure, force, braking, cornering, and striking during the movement of the vehicles. The vibrations occurring as a result of these external effects will also cause stresses, strains, deflections, and surface deformations in the leaf spring system. Hence, it is essential for safety and cost to analyze each of these effects with higher accuracy and to develop the leaf spring system accordingly.

In the literature, the majority of the works have been focused on static analysis, dynamic analysis, material properties, and fatigue prediction. In [2], the authors have realized detailed surface analyses and mechanical tests over a leaf spring system that deformed much earlier than its expected service life. In their work [3], Zhou et al., have designed a new elastic component in order to reduce the working load, ensure the linearity, and regulate the amplitude variations in leaf spring systems. A composite leaf spring system improved by utilizing polymers reinforced with glass fiber has been proposed in [4], and also detailed ANSYS analyses have been realized under the modeling constraints of strains, stresses, and deflection. Odabaşı et al., realized a preliminary comparison among MacPherson, double wishbone, and leaf spring systems in terms of stress and deformation and then represented the results obtained [5]. In another work presented by Abdullah et al. [6], the authors have investigated the reliability assessment based on the predicted fatigue life of a leaf spring under random strain loading. In the composite material-based design analysis realized in [7], the authors dealt with the reduction of the weight in the conventional steel leaf spring by using composite materials. In another work in which the material effect has been analyzed [8], a composite-based mono-leaf spring system has been designed and manufactured to replace existing mono-leaf metal leaf spring structures. In [9], detailed analyses have been realized to analyze the damage levels in composite mono-leaf spring systems, such as the detection, location, and quantification of damage. In work [10], the authors have investigated fiber-reinforced polymeric composite leaf springs instead of a double steel leaf spring. In another work investigating the replacement of existing steel leaf springs with composite leaf springs, the proposed composite leaf spring systems have been tested on the low-velocity impact test rig, and the results obtained have been discussed [11]. In a similar work, a detailed performance comparison between the composite and conventional leaf spring systems was presented by Pozhilarasu and Pillai [12]. In another material analysis-based work [13], the design and experimental analysis of composite leaf spring systems made of glass fiber-reinforced polymer have been realized in terms of the load-carrying capacity, stiffness, and weight savings. Gowd and Goud have presented a detailed ANSYS-based analysis in [14] to determine the safe load of a leaf spring system for which a comfortable and safe drive will be possible. In work [15], a nonlinear elastic leaf spring system model has been developed for use in computer simulations of multibody vehicle systems. In [16,17], the authors compared the performances of composite and steel leaf spring systems in terms of load-carrying capacity, stiffness, and weight savings by taking into account the stress and deflection constraints. The authors in [18] have proposed a composite mono-leaf spring structure, which reduces the weight of the leaf spring system without any reduction in the load-carrying capacity and stiffness and then compared its performance with existing structures. Kong et al., have improved various leaf spring eye designs in their work [19] to attenuate the factors that will lead to failures, such as braking, cornering, and pothole striking occurring during the drive. The authors have proposed a modified steel multi-leaf spring system and then performed its static and fatigue analysis to investigate the load- carrying capacity and life cycles [20]. In their work [21], Shokrieh and Rezaei have proposed an improved leaf spring system geometry in order to obtain a light structure that has the ability to carry static external forces without failure. Finally, a detailed review work on the material selection, design method, and performance investigation of composite leaf spring systems has been presented in [22] by Ke et al.

As seen from the literature review, there are several works related to leaf spring systems consisting of static analysis, dynamic analysis, material properties, and fatigue prediction. However, there is no work in the literature including experimental and simulation-based analyses of the vibration effects occurring in leaf spring systems. To our best knowledge, the 3-axis vibration effects on leaf spring systems have been analyzed in detail and modelled by artificial neural networks for the first time in this work. 

The main contributions of this article to the literature can be summarized as follows:

- The local and regional real-time vibration effects occurring on a steel leaf spring system for different application times and pressure values have been measured experimentally, and the results are presented in the literature. The application time can be defined as the elapsed time between the moments the hydraulic piston first applies pressure to the system and the first cut-off from its contact with the system after applying pressure.
- Two different nonlinear artificial neural network structures (RNANN and CFBANN) have been proposed in order to estimate the vibration data measured from the leaf spring system, and then the detailed analysis results were presented in the literature.
- The experimental system designed and the simulation results obtained will provide a detailed database for leaf spring system manufacturers and researchers, especially in the automotive sector.

The rest of this article is organized as follows: Section 2 presents detailed reviews of both the leaf spring system designed and the ANN structures proposed for the aim of the real-time vibration analysis. In Section 3, the experimental results obtained under different working conditions, and the simulation results obtained for the RBANN and CFBANN structures, are given in detail and then compared with each other. The relevant discussions are presented in Section 4. Finally, the conclusions and future directions are given in Section 5.

## 2. Materials and Methods

**Figure 1:**
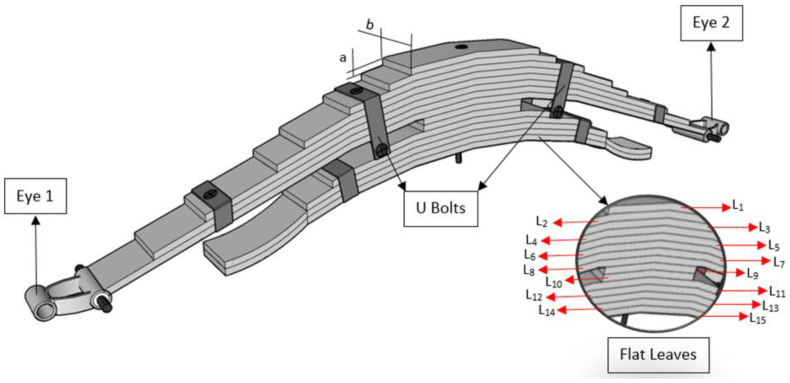
The main components of a leaf spring system.

Leaf spring systems can be designed in different geometries depending on the vehicle type and application area. The main components of a leaf spring system are shown in Figure 1.

**Figure 2:**
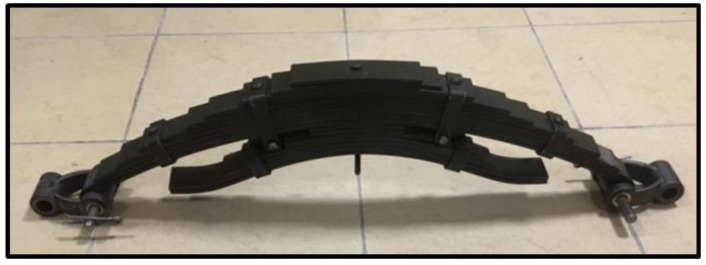
The leaf spring system used in the designed experimental system’s main components of a leaf spring system.

In this work, analyses have been carried out on a steel leaf spring system developed and designed within the scope of [23] by the authors in cooperation with the Daimler Mercedes Aksaray/Turkey factory. This system, which is mostly used in heavy vehicles and construction machinery, has a block structure, including two interconnected but discrete leaf springs, as shown in Figure 2.

As seen from the figure, the main body of the designed leaf spring system includes 10 steel leaf springs. In addition to these 10 leaf springs forming the main body, an additional 7 leaf springs have also been added to strengthen the system and make it more rigid and stable.

The system includes 2 spring eyes, 1 center bolt, 6 clamps, and 2 rubber bushings, and it is also designed to withstand approximately 3 tons of force. The detailed geometric properties of the leaf spring system designed are given in Table 1.

**Figure 3:**
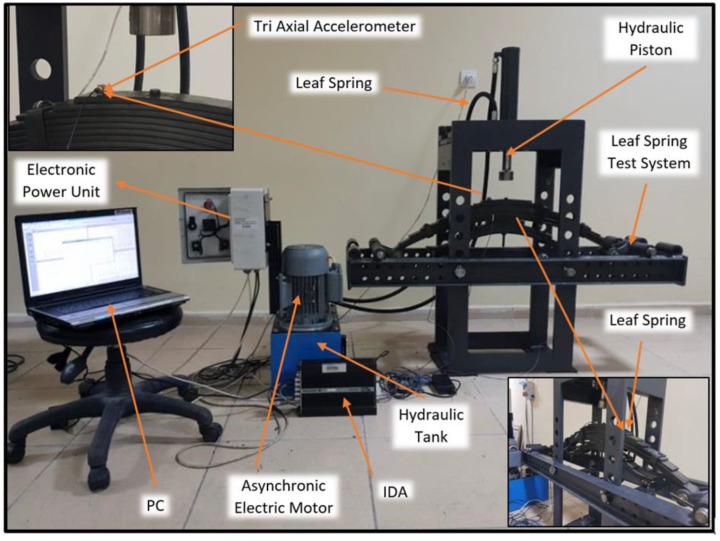
The leaf spring test stand designed for the vibration analysis.

The detailed representation of the whole experimental system, including the leaf springs, hydraulic system components, electronic unit, test stand, and 3-axial accelerometer is given in Figure 3. In order to perform the vibration analysis, a Brüel and Kjaer 4524 B 001 type 3-axial accelerometer was used. Furthermore, to make the data obtained from the sensors more meaningful, a Brüel and Kjaer 3560 B-type intelligent data acquisition (IDA) tool was used. The 3-axial accelerometer was placed on the uppermost leaf spring so as to be in the same direction as at the point the pressure was applied, as shown in Figure 3.

The cylinder diameter used in the designed system is preferred as  D=6cm. Accordingly, the total area of the cylinder was obtained as $\;A=\pi {\left(\frac{D}{2}\right)}^{2}≅28.26{\mathrm{cm}}^{2}$. According to these design criteria, the force values corresponding to each pressure value were measured as follows (see Table 2).

### 2.1. Artificial Neural Network

**Figure 4:**
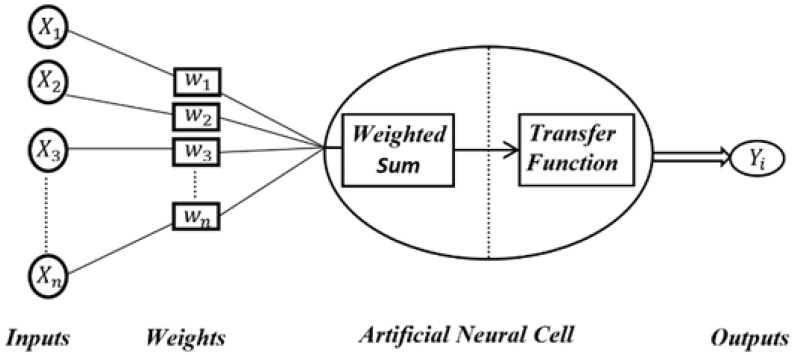
Structure of an artificial neural network.

The ANNs, inspired by the electrical activities of the brain and nervous system, are one of the most commonly preferred parallel computational systems in the literature due to their estimation ability with high accuracy [24]. The general structure of an ANN is represented in Figure 4.

In ANN networks, the $w_{i}$ weight values of the network connections are optimized after each feedforward so as to minimize the mean squared error (*MSE*) given in the following equation and represent the difference between the desired and actual outputs [25]:

(1) $$MSE=\frac{1}{N}{\sum_{i=1}^{MI}\left(Y_{Desired}-Y_{Actual}\right)}^{2}$$

where, MI is the maximum iteration number, $Y_{Desired}$ is the desired response, which corresponds to the experimental results, and, finally, the $Y_{Actual}$ represents the estimation result produced by the ANN at the relevant iteration.

In this work, two ANN structures of the Radial Basis Artificial Neural Network (RBANN) and Cascade-Forward Back-Propagation Artificial Neural Network (CFBANN) have been proposed to model the real-time vibration characteristics occurring on a leaf spring system. Each ANN structure proposed contains two neurons for the input parameters and three neurons for the output parameters. While the input neurons correspond to the application time and pressure value parameters, the output neurons correspond to the vibration values obtained from the 3-axial accelerometer.

### 2.2. Radial Basis Artificial Neural Network

**Figure 5:**
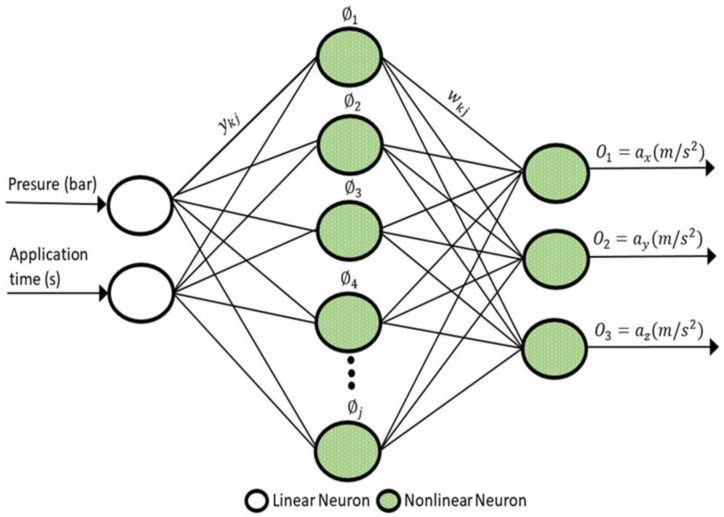
The RBANN structure used in the simulations.

The RBANN, which was proposed by Broomhead and Lowe [26], has an effective feedforward network structure based on the Gaussian activation function. The RBANN network structure consists of an input layer, hidden layer, and output layer, as in the ANN. The schematic structure of the proposed RBANN is shown in Figure 5.

In this structure, a nonlinear transformation is applied from the input layer to the hidden layer by using a Gaussian activation function given in Equation (2). On the other hand, a linear transformation is performed between the hidden layer and output layer:

(2) $$\phi_{j}=\exp\left[-{\left(\frac{\left\|x-c_{j}\right\|}{\sigma }\right)}^{2}\right]$$

where x is the input vector, $c_{j}$ represents the centre of the jth Gaussian function, σ>0 determines the spread constant, and, finally, the $\left\|x-c_{j}\right\|$ notation denotes the Euclidean distance between the x and $c_{j}$ vectors. In this structure, $\phi_{j}$ can also be defined as the activation level of the jth hidden neuron. Hence, the output of the neuron, k, will be obtained, as given in Equation (3):

(3) $$O_{k}=\sum_{j=1}^{J}w_{kj}.\phi_{j}$$

where J represents the number of hidden layers, and $w_{kj}$ is the weight value between the kth  output neuron and jth hidden neuron. Finally, the spread constant parameter has a direct effect on the global performance of the ANN structure. If the σ value is not chosen appropriately, the ANN will have difficulty efficiently approximating fast-changing functions. In order to find the most appropriate value of the spread constant parameter, the simulations based on test data (30% of the experimental data) were performed for the 0.1 step sizes in the range (0,1], in line with the problem structure and the literature [27]. From the MSE values obtained for each possible σ value, it is seen that the optimal value of σ is 0.5. 

### 2.3. Cascade-Forward Back-Propagation Artificial Neural Network

The back propagation (BP) learning algorithm is basically based on the philosophy of feedback of the total error in a feedforward network structure to the neurons in the hidden layer for the purpose of increasing the success of training [28]. The effect of a neuron on related output is directly associated with the weight value of that neuron. 

In order to update the weight values between the input and hidden layers, the BP algorithm uses the following equation:

(4) $$\Delta w_{ij}\left(t\right)=-\eta \;\frac{\partial E_{2}\left(t\right)}{\partial w_{ij}}+\alpha .\Delta w_{ij}\left(t-1\right)$$

Similarly, the weight values between the hidden layer and the output layer are updated by using the equation given below:

(5) $$\Delta w_{in}\left(t\right)=-\eta \;\frac{\partial E_{1}\left(t\right)}{\partial w_{jn}}+\alpha .\Delta w_{jn}\left(t-1\right)$$

where η and α represent the learning rate and momentum factor, respectively, $E_{1}$ is the error between the experimental and actual outputs, and, finally, $E_{2}$ can be defined as the propagation error between the input layer and hidden layer. The values of the η and α parameters have been taken as 0.25 and 0.4, respectively.

**Figure 6:**
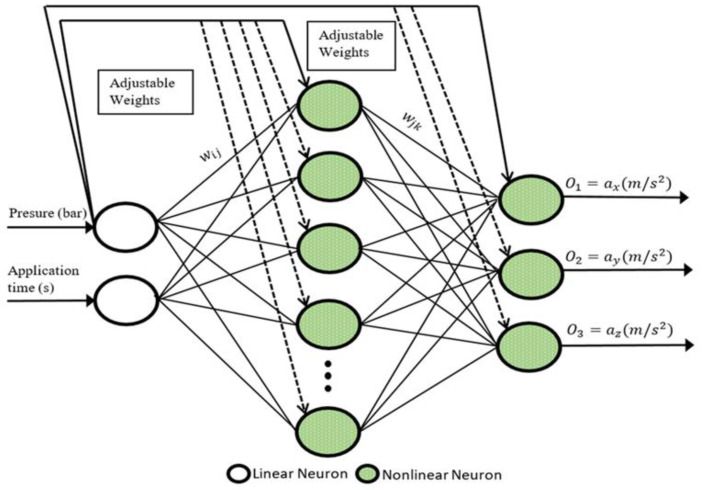
The CFBANN structure used in the simulations.

In this work, a cascade-forward back-propagation (CFB) learning algorithm, in which the input values are linked to all of the layers, was used. The schematic representation of the CFBANN algorithm proposed is given in Figure 6. In the CFBANN network structure, the relationship between the input and output layers can be appropriately established by using two or more layers and selecting a sufficient number of neurons. The most commonly preferred transfer functions in an ANN-based estimation are the logarithm sigmoid (logsig), tangent sigmoid (tansig), and linear (pureline) transfer functions. In this work, the tangent sigmoid (tansig) function, given in Equation (6), has been preferred as a transfer function in both RBANN and CFBANN networks.

(6) $$f\left(x\right)=\frac{1}{1+e^{-x}}$$

Finally, the CFBANN-based training results demonstrate that the optimum hidden neuron number for the CFBANN was found as 50. 

## 3. Results

In this work, an experimental measurement system was improved to measure real-time local vibrations occurring on a leaf spring system under different application times and pressure values. In the experimental phase of the work, the real-time vibration data occurring on the leaf spring system were experimentally measured with a Brüel and Kjaer 4524 B 001 3-axial accelerometer. In the simulation phase carried out by using the MATLAB Neural Network Tool (MATLAB NNTool), these real-time vibration data were analyzed with nonlinear ANN structures under different application times and pressure values.

**Figure 7:**
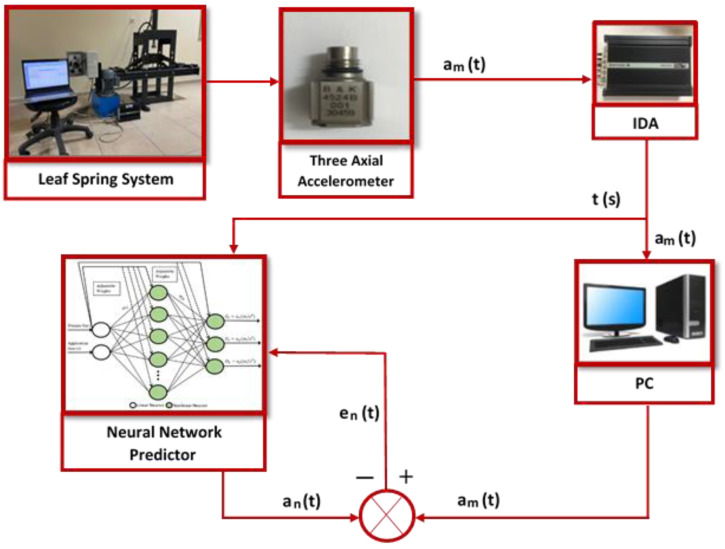
The block diagram of the whole system and consisting of both experimental and simulation approaches.

The experimental measurements were separately realized for the application times of 11, 14, and 20 s (sec.), and data were taken with a sensitivity of 6% of a second in each application time. For each application time, the leaf spring system was exposed to the pressure values of 6.25, 18.75, and 37.5 bar, which corresponds to low, medium, and high-pressure values, respectively. The data were collected throughout the relevant application time from the beginning to the end. Finally, the experiments were repeated three times for each application time because it was observed that the results produced showed similar characteristics after three repetitions. The block diagram of the whole system, consisting of both the experimental and simulation approaches, is shown in Figure 7. In this figure, $a_{m}(t)$ is a function that varies depending on the 3-axial acceleration measurement results (a_x_, a_y_, and a_z_) obtained from the *x*, *y*, and *z* axes. Similarly, $a_{n}(t)$ represents the 3-axial acceleration measurement results predicted by the neural network predictor. t(s) represents the time variation, and, finally, $e_{n}(t)$ represents the time-dependent MSE error values.

The ANNs use training and test phases for the purpose of improving an effective model for the problem. In this work, and consistent with the general approach in the literature, a randomly selected 70% of the experimental data were used for the training phase, while the remaining data were used for the test phase. The application times preferred and the proportional distribution of data in the simulations are given in Table 3.

The four results given below can be generalized for all of the experimental and simulation results:

(i) The peak waves represent the vibration values occurring on the leaf spring system when the hydraulic piston first cut-off its contact with the system after applying pressure.
(ii) Since the hydraulic piston moves along the y-axis, it is seen that the highest peak values occur on the *y*-axis.
(iii) The duration of the vibrations occurring on the leaf spring system increase or decrease proportionally with the application time.
(iv) All simulations were realized for a randomly selected three consecutive pulse durations within the total response obtained in the experimental results.

**Figure 8:**
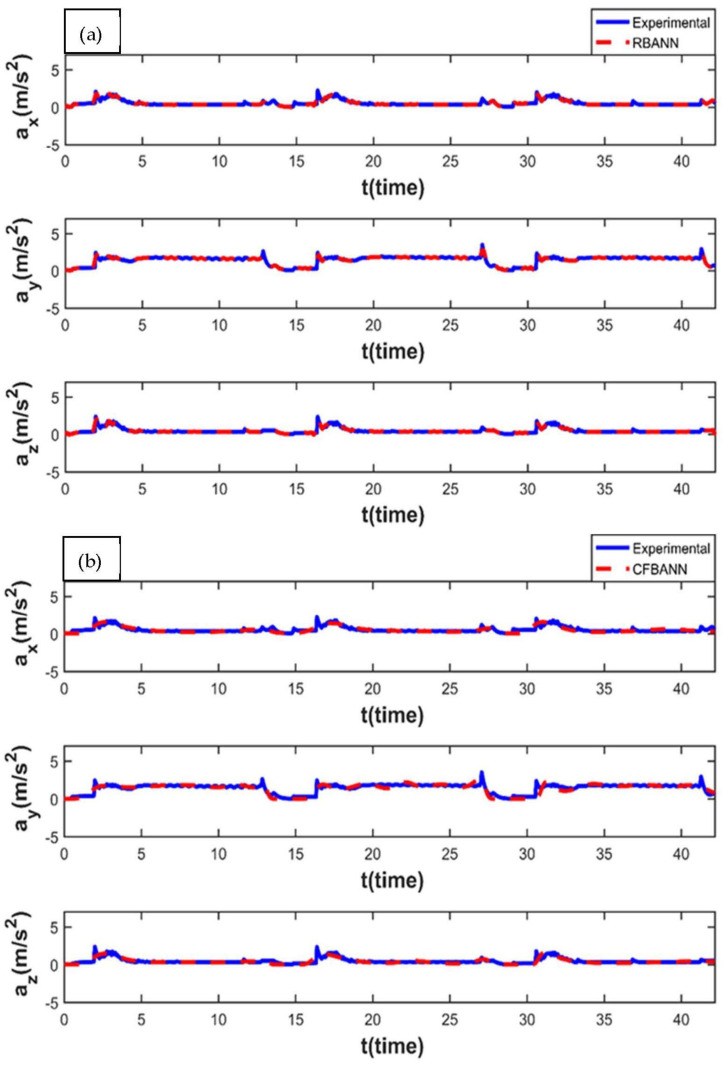
Experimental and ANN results obtained for the working condition of 11 s and 6.25 bar: (**a**) RBANN; (**b**) CFBANN.

The experimental and ANN results obtained for the working condition of 11 s and 6.25 bar are given in Figure 8. It is observed that the weak peak waves appeared on the system as expected for 6.25 bar, which is a low-pressure value. It can also be stated that RBANN produces similar but a bit better results in terms of learning performance when compared to CFBANN.

**Figure 9:**
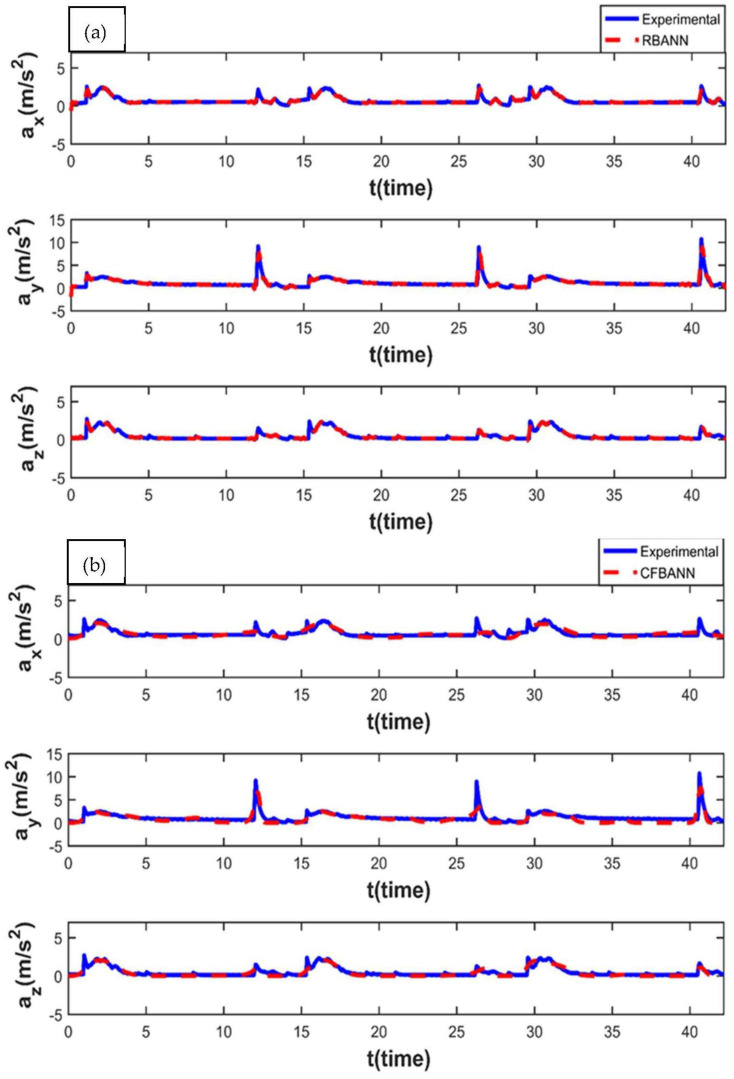
Experimental and ANN results obtained for the working condition of 11 s and 18.75 bar: (**a**) RBANN; (**b**) CFBANN.

The vibration results obtained when the application time remained at 11 s and the pressure value was increased to 18.75 bar are given in Figure 9. It is seen that the RBANN shows better performance than the CFBANN in estimating the vibration characteristics of the leaf spring system, especially at the peak values and *y*-axis.

**Figure 10:**
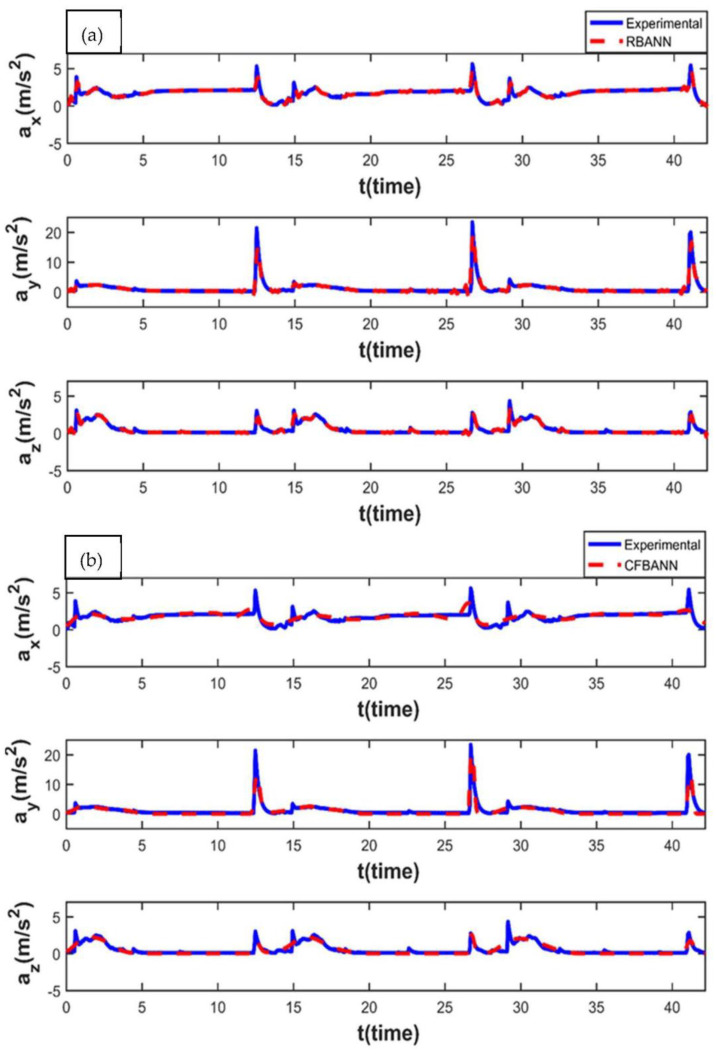
Experimental and ANN results obtained for the working condition of 11 s and 37.50 bar: (**a**) RBANN; (**b**) CFBANN.

In Figure 10, the results obtained for 37.5 bar, which was determined as the highest pressure value in the experimental system, were given in the 11 s application time. Although the convergence performances of both learning algorithms to the experimental results are similar, it can be expressed that the RBANN algorithm shows better performance, especially for peak waves.

**Figure 11:**
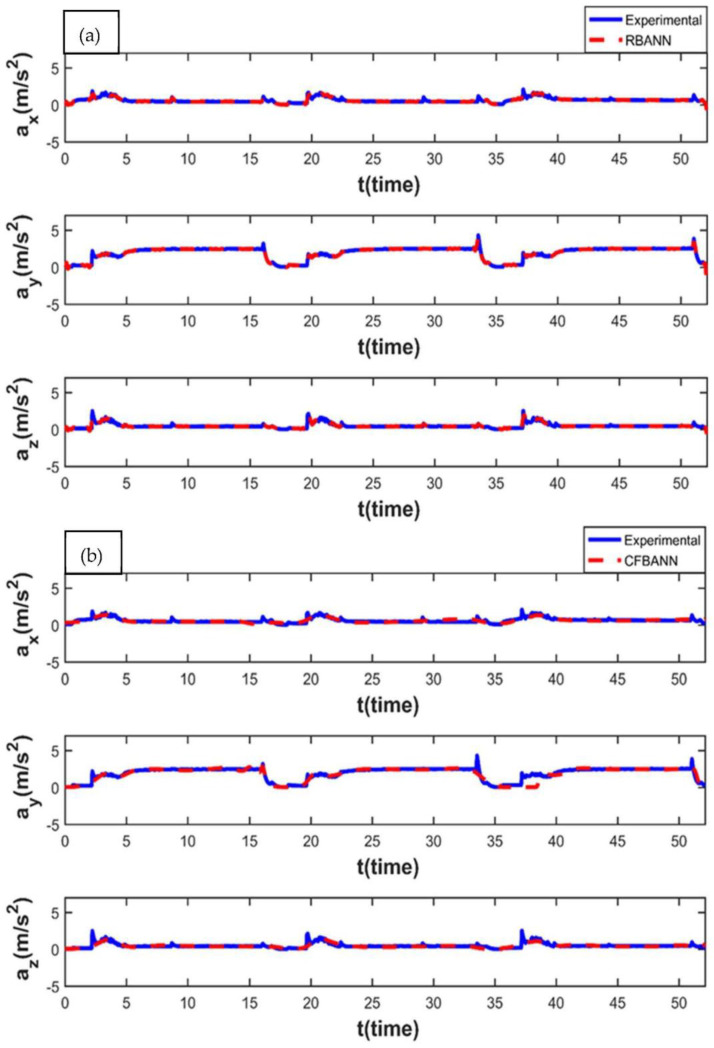
Experimental and ANN results obtained for the working condition of 14 s and 6.25 bar: (**a**) RBANN; (**b**) CFBANN.

Figure 11 shows the experimental and ANN results obtained under the condition of 14 s and 6.25 bar. From the simulation results obtained, it is seen that the RBANN algorithm successfully converges to the characteristics of the experimental results. On the other hand, it is seen that small deviations occur from the experimental results in some regions when the CFBANN algorithm is used.

**Figure 12:**
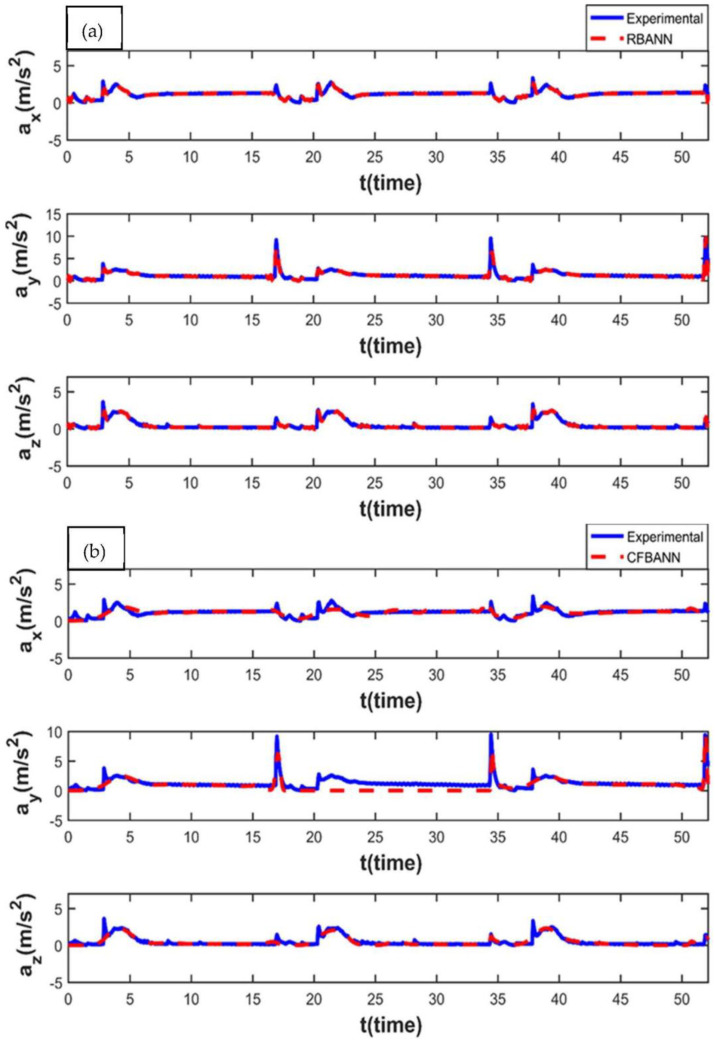
Experimental and ANN results obtained for the working condition of 14 s and 18.75 bar: (**a**) RBANN; (**b**) CFBANN.

The vibration characteristics of the leaf spring system for 14 s and 18.75 bar parameter values and the ANN results according to these parameter values are given in Figure 12. It is seen that the modeling performance of the CFBANN algorithm is quite low at the y-axis, in which the hydraulic piston moves along. On the other hand, it is also seen that the RBANN has the capability of estimating the experimental results with higher accuracy than the CFBANN.

**Figure 13:**
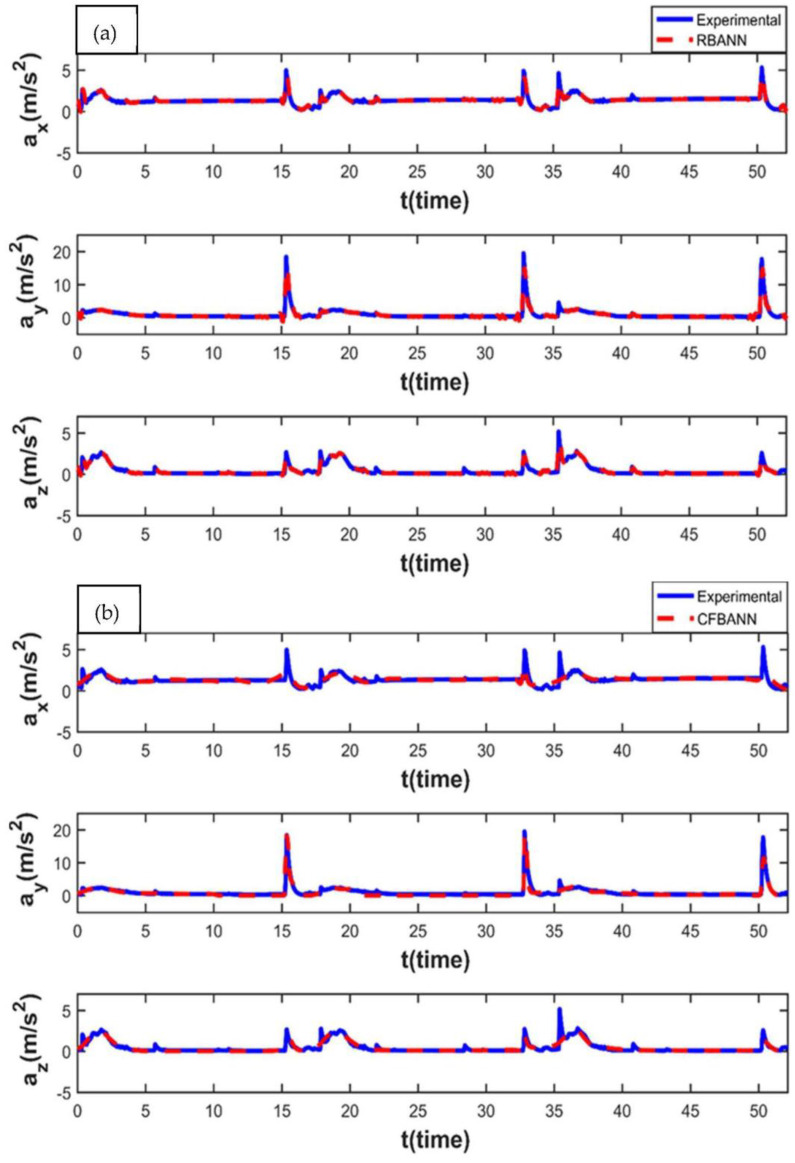
Experimental and ANN results obtained for the working condition of 14 s and 37.5 bar: (**a**) RBANN; (**b**) CFBANN.

The results obtained for 14 s and 37.5 bar represent that when the pressure value is increased to six times the initial pressure, the peak values in the measurements also increase proportionally. When the results given in Figure 10 and Figure 13 are compared, it can be stated that increasing application times cause an increase in the duration of the vibration at the same time. Finally, the learning performance of the CFBANN seems a bit worse, especially in the x and z axes, when compared to the RBANN.

**Figure 14:**
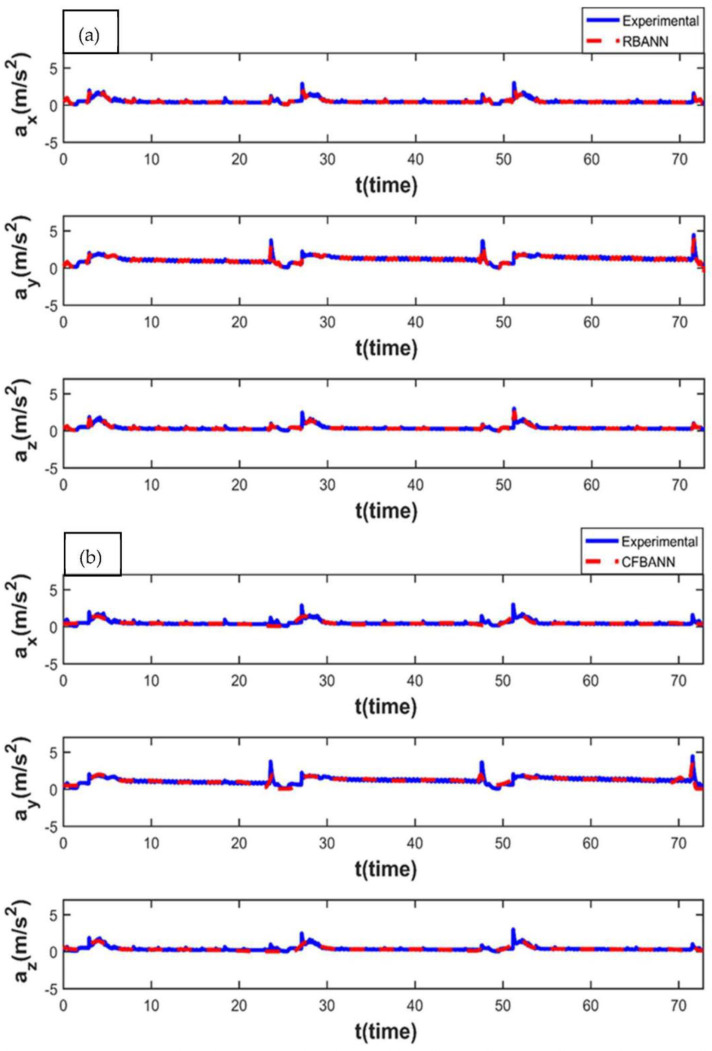
Experimental and ANN results obtained for the working condition of 20 s and 6.25 bar: (**a**) RBANN; (**b**) CFBANN.

When the application time is increased to its highest value of 20 s, the results obtained for the 6.25 bar pressure value are shown in Figure 14. It can be observed from the figures that both learning algorithms are able to estimate the experimental results successfully. However, it can also be expressed that the RBANN produces better results than the CFBANN in converging to the peak values.

**Figure 15:**
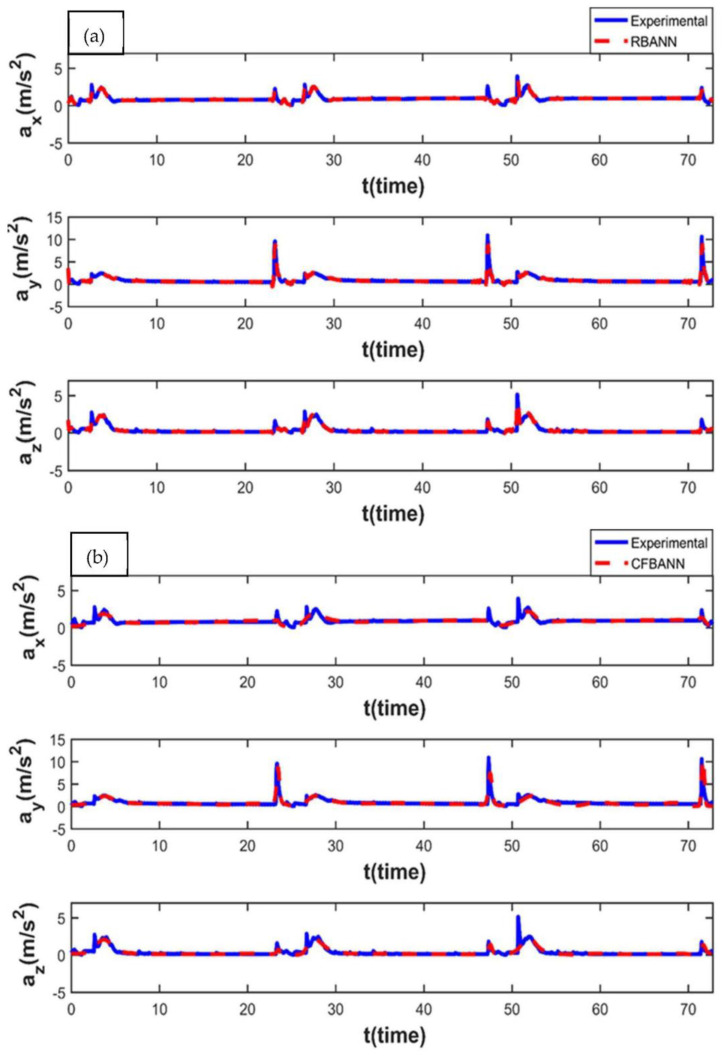
Experimental and ANN results obtained for the working condition of 20 s and 18.75 bar: (**a**) RBANN; (**b**) CFBANN.

The vibration characteristics of the leaf spring system for 20 s and 18.75 bar values and the ANN results, according to these parameter values, are given in Figure 15. It can be stated from the figures that RBANN shows better performance than the CFBANN in estimating the vibration characteristics of the leaf spring system, especially at the peak values at the x and z axes.

**Figure 16:**
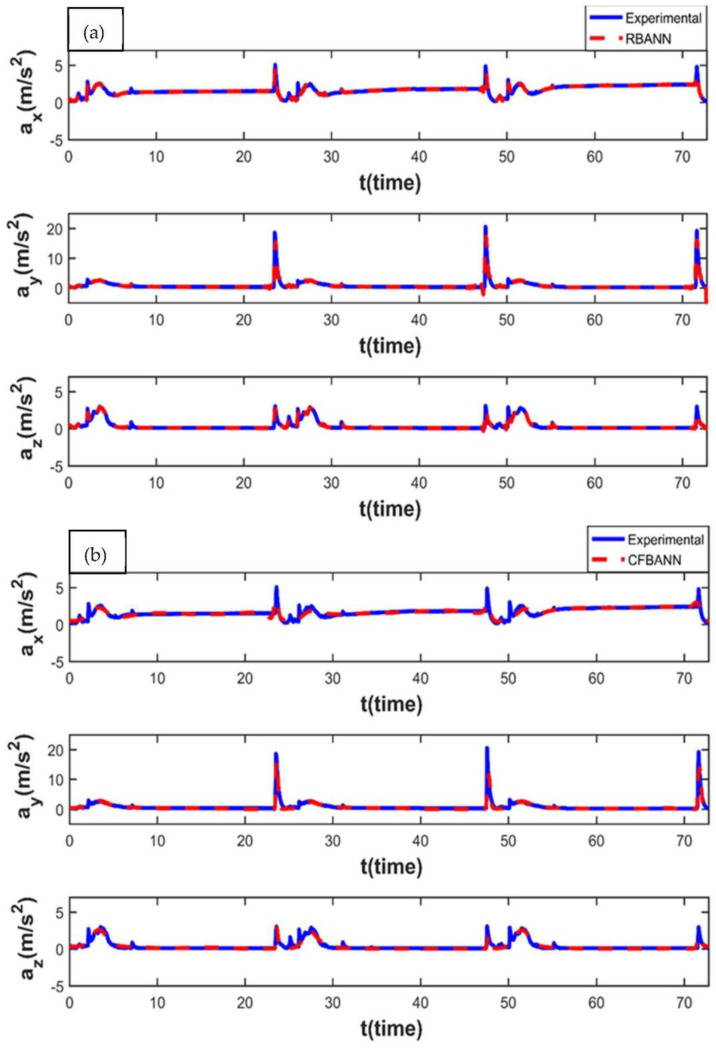
Experimental and ANN results obtained for the working condition of 20 s and 37.5 bar: (**a**) RBANN; (**b**) CFBANN.

When these results, obtained at the highest pressure and application time values, are compared with the previous relevant results, it is seen that the RBANN and CFBANN produce similar results as in the previous application times. Moreover, it can also be seen from Figure 16 that the maximum vibration durations and accelerating values are reached under this working condition.

From the simulation results given so far, it has been observed that the performance of the CFBANN algorithm in estimating the experimental results obtained along the y-axis, in which the hydraulic piston moves along, was insufficient for some of the working conditions. It is also seen that the performance of the CFBANN algorithm in estimating the peak values occurring on the leaf spring system, each of which represents an instantaneous vibration value, was generally insufficient. Finally, it can be stated that the RBANN algorithm exhibits a more robust behavior under all of the working conditions and estimates the experimental results with higher accuracy when compared to the CFBANN algorithm. On the other hand, the experimental results prove that the amount of pressure applied to the leaf spring system has a direct effect on the magnitude of the vibrations occurring on the *x*, *y*, and *z* axes. 

In order to compare the performances of the created RBANN and CFBANN structures in terms of the root-mean-squared error (RMSE) values reached, the relevant error values obtained in the acceleration measurements (a_x_, a_y_, and a_z_) for the *x*, *y*, and *z* axes in different working conditions are separately given in Table 4. As seen from the table, the RBANN structure created produces better results for all of the working conditions when compared to that of the CFBANN.

In order to analyze the statistical performances of the ANN network structures improved, coefficient of determination (R-squared or R^2^) analyses were also performed. In the context of regression, R^2^ is a statistical measure of how close the regression line approximates the actual data. The R^2^ analysis is, therefore, important when a statistical model is used to predict future outcomes.

The mathematical expression of R^2^ can be given as follows:

(7) $$R^{2}=1-\frac{\sum {\left(y_{i}-{\hat{y}}_{i}\right)}^{2}}{\sum {\left(y_{i}-\tilde{y}\right)}^{2}}$$

where $\;y_{i}$ represents the experimentally obtained values, ${\hat{y}}_{i}$ are the values calculated from the regression equation, and, finally, $\tilde{y}$ can be defined as the mean value of the experimental data. The R^2^ parameter takes values in the interval of [0,1], and the higher R^2^ values prove the success of the model. As seen in Table 5, the RBANN produces a higher statistical performance in terms of R^2^ when compared to the CFBANN. Consequently, it can be emphasized from the statistical analysis that the stability and robustness of the RBANN are quite adequate in improving the ANN structures to model the experimental data.

## 4. Discussion

Computer-aided detection and modeling of disruptive factors, such as stress, strain, deflection, and deformation, occurring on mechanical systems caused by vibrations, have become an active area of research in recent years. The detection and modelling of these external effects with high accuracy are crucial for the design of robust systems. ANNs, as one of the most important computer-aided approaches, present high performance in terms of the accurate estimation of the disruptive factors in real-time analysis.

In this work, the simulations were realized by using the RBANN- and CFBANN-based ANN structures. From the results obtained for all of the working conditions, it was seen that the proposed RBANN structure has an effective performance in analyzing the vibration characteristics of a leaf spring system. On the other hand, it was also seen that the CFBANN produces similar but a bit worse results than the RBANN at low pressures and it presents an insufficient performance especially in the estimation of high amplitude peak values representing instantaneous vibrations. From the simulation results obtained for the ANN-based estimation of the experimental results, it is seen that the RBANN produces more effective results than the the CFBANN under all of the working conditions. Furthermore, the RMS error values reached prove that the RBANN-based ANN structures provide more effective and stable solutions in estimating the real-time system responses. Finally, in the sense of experimental vibration characteristics, it can be concluded that the amount of pressure applied, and the application time of it, directly affects the vibration characteristics for all three axes 

## 5. Conclusions

In this work, the RBANN and CFBANN algorithms, which are among the most effective ANN structures in the literature, were used for the estimation of the real-time 3-axial vibration data obtained experimentally from a leaf spring system. From the simulation results, it was observed that the performances of the algorithms, in terms of the estimation and RMS error value, are close to each other. However, it was also seen that the RBANN produced a bit more of a robust behavior under all of the working conditions, especially for the peak values, each of which represents an instantaneous vibration value. Consequently, it is clearly seen from the results obtained that the performance of the RBANN and CFBANN algorithms, in terms of estimation, are too similar, and they can efficiently be used in the estimation of real-time responses.

In future research, firstly, the experimental studies will be focused on the measurement of the forces occurring in different regions of a leaf spring system. The experimental studies will be carried out by using a 3-axial KISTLER force sensor, and the ANN-based modelling of the experimental results will also be performed. In addition, the disruptive factors, such as stress, strain, deflection, and deformation, occurring due to the external forces the leaf spring system exposed will also be analyzed in detail.

## Figures and Tables

**Table 1:** Geometric properties of the leaf spring system designed.

Geometric Properties		
Length of flat leaves, L	**Upper Leaves**	**Lower Leaves**
	L_1_ = 200 mm	L_9_ = 150 mm
	L_2_ = 260 mm	L_10_ = 150 mm
	L_3_ = 365 mm	L_11_ = 235 mm
	L_4_ = 485 mm	L_12_ = 405 mm
	L_5_ = 555 mm	L_13_ = 475 mm
	L_6_ = 725 mm	L_14_ = 675 mm
	L_7_ = 830 mm	L_15_ = 675 mm
	L_8_ = 937 mm	
Width of each flat leaf (b)	0.8 cm	
Thickness of each flat leaf (a)	5.786 cm	
Distance between U-bolts	980 mm	
Outer Diameter of each Eye	53 mm	
Inner Diameter of each Eye	37 mm	

**Table 2:** The force values that occurred according to variable pressure values.

Pressure (bar)	Force (kg)
6.25	176.625
18.75	515.745
37.5	1059.75

**Table 3:** The force values that occurred according to the variable pressure values.

Application Time (seconds)	Number of Experimental Data Used	Number of Training Data Used	Number of Test Data Used
11	4224	2957	1267
14	5220	3654	1566
20	7284	5099	2185

**Table 4:** Experimental working conditions and RMSE values obtained in the *x*, *y*, and *z* axes.

Working Conditions		RMSEs					
Application Time (seconds)	Pressure Value (bar)	RBANN			CFBANN		
		a_x_	a_y_	a_z_	a_x_	a_y_	a_z_
11	6.25	0.0909	0.1079	0.0977	0.2157	0.2916	0.1981
	18.75	0.1148	0.3197	0.1109	0.3117	0.7004	0.2544
	37.5	0.1755	0.7632	0.1648	0.4394	1.0480	0.3158
14	6.25	0.0729	0.1063	0.0973	0.1753	0.3372	0.2024
	18.75	0.1144	0.3252	0.1314	0.2956	0.8030	0.2498
	37.5	0.1606	0.5473	0.1487	0.3677	0.6795	0.2876
20	6.25	0.0887	0.1030	0.0836	0.1994	0.2556	0.1614
	18.75	0.0979	0.2699	0.1321	0.2287	0.5084	0.2465
	37.5	0.1150	0.5470	0.1116	0.2780	0.7374	0.2306

**Table 5:** Experimental working conditions and R2 values obtained in the *x*, *y*, and *z* axes.

Working Conditions		R^2^					
Application Time (seconds)	Pressure Value (bar)	RBANN			CFBANN		
		a_x_	a_y_	a_z_	a_x_	a_y_	a_z_
11	6.25	0.9380	0.9680	0.9230	0.6910	0.8150	0.7250
	18.75	0.9530	0.9060	0.9660	0.6740	0.6540	0.8480
	37.5	0.9300	0.8960	0.9490	0.6630	0.8600	0.8380
14	6.25	0.9460	0.9840	0.9090	0.6880	0.8690	0.6100
	18.75	0.9380	0.8790	0.9570	0.6120	0.8460	0.8920
	37.5	0.9140	0.9110	0.9540	0.6380	0.8730	0.8700
20	6.25	0.9200	0.9410	0.9250	0.6250	0.6630	0.7330
	18.75	0.9350	0.9110	0.9430	0.6460	0.7520	0.8010
	37.5	0.9630	0.8980	0.9700	0.7810	0.8210	0.8730

## Data Availability

Not applicable.
